# Impacts of plasmonic nanoparticles incorporation and interface energy alignment for highly efficient carbon-based perovskite solar cells

**DOI:** 10.1038/s41598-022-09284-9

**Published:** 2022-03-30

**Authors:** MirKazem Omrani, Reza Keshavarzi, Mojtaba Abdi-Jalebi, Peng Gao

**Affiliations:** 1 Department of Physics, University of Isfahan, 81746-73441 Isfahan, Iran; 2 Department of Chemistry, Catalysis Division, University of Isfahan, 81746-73441 Isfahan, Iran; 3 Institute for Materials Discovery, University College London, Malet Place, London, WC1E 7JE UK; 4 Xiamen Key Laboratory of Rare Earth Photoelectric Functional Materials, Fujian Institute of Research On the Structure of Matter, Chinese Academy of Sciences, Xiamen, 361021 Fujian People’s Republic of China

**Keywords:** Electronic properties and materials, Nanoparticles, Electronics, photonics and device physics, Solar cells

## Abstract

This work utilizes a realistic electro-optical coupled simulation to study the (i) impact of mesoporous TiO_2_ removal; (ii) the embedding of Ag@SiO_2_ and SiO_2_@Ag@SiO_2_ plasmonic nanoparticles; (iii) utilization of solution-processed inorganic p-type copper(I) thiocyanate (CuSCN) layer at the perovskite/carbon interface; and (iv) the increase of the work function of carbon electrodes (via incorporation of suitable additives/binders to the carbon ink) on the performance of carbon-based PSCs. Removal of mesoporous TiO_2_ increased the power conversion efficiency (PCE) of the device from 14.83 to 16.50% due to the increase in exciton generation rate and charge carriers’ mobility in the vicinity of the perovskite-compact TiO_2_ interface. Subsequently, variable mass ratios of Ag@SiO_2_ and SiO_2_@Ag@SiO_2_ plasmonic nanoparticles are embedded in the vicinity of the perovskite-compact TiO_2_ interface. In the optimum cases, the PCE of the devices increased to 19.72% and 18.92%, respectively, due to light trapping, scattering, and strong plasmonic fields produced by the plasmonic nanoparticles. Furthermore, adding the CuSCN layer remarkably increased the PCE of the device with a 0.93% mass ratio of Ag@SiO_2_ nanoparticles from 19.72 to 26.58% by a significant improvement of V_oc_ and FF, due to the proper interfacial energy band alignment and the reduction of the recombination current density. Similar results were obtained by increasing the carbon work function, and the cell PCE was enhanced up to 26% in the optimal scenario. Our results pave the way to achieve high efficiencies in remarkably stable printable carbon-based PSCs.

## Introduction

Over the past decade, perovskite solar cells have become a promising candidate as a future source of energy. The unique optical and electrical properties of perovskites, such as high absorption coefficient, direct bandgap, long carrier diffusion length, and low exciton binding energy, have led to rapid growth and a significant increase in the device efficiency from 3.8 to > 25%^1,2^. However, most of the high-efficiency perovskite solar cells are fabricated with expensive organic materials (e.g., 2,2′,7,7′-tetrakis-(*N*,*N*-di-4-methoxyphenylamino)-9,9′-spirobifluorene (spiro-OMeTAD)) as the hole transfer layer (HTL) and noble metal (e.g., Au) as the electrode, which is deposited by thermal evaporation in a vacuum environment. This not only increases the cost of production but also reduces the stability of the device due to the degradation because of the mobile behavior of Au contact at 70 °C and the poor stability of Spiro-OMeTAD^3,4^.

In recent years, HTL-free carbon-based perovskite solar cells have attracted much academic and industrial attention for their outstanding stability with lower production costs^5–12^. In this case, when a carbon-based material is replaced with the metal contact and the HTL, the non-encapsulated device exhibits excellent stability in an ambient environment^13^. However, the efficiency of carbon-based devices is significantly lower than their counterparts equipped with HTL/metal back contact, where the highest efficiency of HTL-free carbon-based perovskite solar cells is ~ 15%^14^.

In general, the interface of the perovskite photoactive layer and the electron transfer layer (ETL) (which often includes the compact and mesoporous TiO_2_) plays a crucial role in achieving high efficiency^15,16^. The mesoporous structure acts as a pathway for electron transfer by increasing the contact surface between the perovskite and TiO_2_^17^. However, the presence of isolated TiO_2_ nanoparticles can lead to charge accumulation and increased recombination due to the trapping of electron carriers and the low mobility of carriers in the perovskite-filled mesoporous structure. This, in turn, affects hysteresis behavior in measuring the efficiency and instability of the device due to the ion migration^18^. Therefore, providing a carbon-based planar structure by removing mesoporous TiO_2_ can not only reduce the device's hysteresis behavior^19^ but also take another step toward commercializing perovskite-based solar cells by reducing the cost of mass production.

One of the most effective approaches to increase the efficiency of carbon-based perovskite solar cells is to use plasmonic nanoparticles within the device structure^20–26^. Under light illumination, metal nanoparticles create strongly localized fields around them during the coherent oscillations of their conducting electrons^27,28^, which can not only increase the photon flux inside the perovskite as a secondary light source but also increase the generation rate of free carriers by reducing the exciton binding energy^29^. In addition, the light scattering by resonated nanoparticles can increase the absorption via enlarging of the light pathway, even at thin perovskite thicknesses^30^. On the other hand, plasmonic nanoparticles generate hot electrons during plasmon resonance which can be directly injected into their surrounding perovskite environment and increase photo-generated electrons mobility by filling the trap states^31^.

To take advantage of local surface plasmon resonance (LSPR) effects, metal nanoparticles must be added to the photoactive layer. First, however, a capping layer over the metallic nanoparticles must be used due to the corrosive properties of CH_3_NH_3_PbI_3_, which lead to the decomposition of noble metals^32^. Then, heating the nanoparticles metallic surface during plasmon resonance will lead to the recombination of excitons at their surface^33^. Here, the usage of a thin layer of SiO_2_ as a capping layer not only stabilizes the noble metal nanoparticles against thermal and chemical interactions but also creates the slightest manipulation in the LSPR properties of the nanoparticles^21^. The presence of a dielectric coating layer can attenuate the plasmonic properties of the nanoparticles. In some studies^21,34,35^, to improve the plasmonic effects of nanoparticles, triple core–shell nanostructures with a metal nanoshell sandwiched between the dielectric outer shell and core (e.g. SiO_2_@Ag@SiO_2_ and TiO_2_@Au@TiO_2_) have been proposed, which can produce better LSPR properties thanks to their plasmon hybridization mechanism.

Herein, we improve the performance of HTL-free carbon-based planar perovskite solar cells using plasmonic NPs. Firstly, the photovoltaic measurements of the fabricated HTL-free carbon-based perovskite solar cell have been utilized for simulation and extract the electrical parameters via fitting its corresponding *J-V* characteristics. Then, a detailed analysis of the device performance in the presence of mesoporous TiO_2_ and planar structure has been presented using electro-optical coupled simulation regimes. Next, the effect of realistic embedding of Ag@SiO_2_ and SiO_2_@Ag@SiO_2_ nanoparticles with different concentrations on the optical and electrical performance of the carbon-based planar perovskite solar cell has been investigated. Finally, since the presence of a carbon electrode (work function of ~ 5 eV) as the non-ideal anode limits the device efficiency, for increasing the transfer rate of hole carriers and blocking the electron carriers, we investigate the effects of adding a copper(I) thiocyanate (CuSCN) interlayer at the perovskite-carbon interface and increasing the working function of carbon electrode on the performance of carbon-based perovskite solar cells.

## Results and discussion

**Figure 1:**
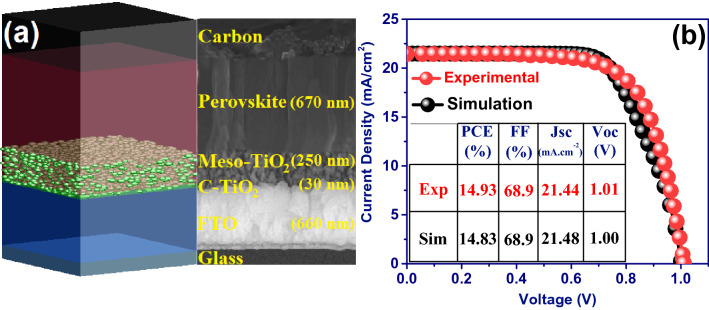
(**a**) Device configuration used for simulation and cross-section image of the experimental HTL-free carbon-based perovskite solar cell. (**b**) The photocurrent density–voltage (J-V) curves from experimental and simulation validation of the perovskite solar cell. J-V characteristics of the devices have been measured under one sun simulated illumination.

The structure of the fabricated and modeled device is Glass/FTO/Compact TiO_2_/Mesoporous TiO_2_/CH_3_NH_3_PbI_3_/Carbon (see Fig. 1a). Here, the mesoporous TiO_2_ is considered inside the perovskite photoactive layer. In other words, the pore sites of the mesoporous TiO_2_ are filled with perovskite material. In the simulated structures, mesoporous TiO_2_ is made of deformed TiO_2_ nanospheres with a size distribution of 7–10 nm that can overlap with each other and randomly distribute within an equivalent thickness of ~ 250 nm with a porosity of ~ 39% (Fig. 1a). This modeling is based on the results of FESEM and ellipsometry analyzes of the fabricated solar cell^40^. Accurate modeling of the structure of the mesoporous TiO_2_ is crucial because it plays a vital role in the distribution of the field profile within the perovskite layer. This directly affects the exciton distribution, generation, and recombination rates of charge carriers along with the photoactive layer. Essential details considered in this modeling have not been reported in any of the previously published works.

Figure 1b shows the measured current–voltage curves from the experimental device compared to the simulation results, wherein the simulation results are in complete agreement with the experimental data. The power conversion efficiency (PCE) of 14.93% and 14.83% for the HTL-free carbon-based perovskite solar cell has been measured and obtained from the simulation, respectively. Furthermore, all the electrical characteristics obtained from the simulation (V_oc_, J_sc_, FF, and PCE) are in good agreement with the experimental data, which shows that the modeling performed in this work can explain the experimental optical-electrical characteristics. Therefore, it provides a good description of the device performance.

**Figure 2:**
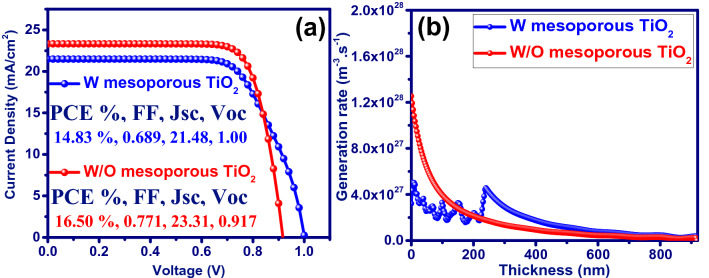
(**a**) J–V characteristics of the device and (**b**) Spatial profile of photo-generated charges in CH_3_NH_3_PbI_3_ photoactive layer with (W) and without (W/O) incorporation of mesoporous TiO_2_.

The first perovskite solar cell manufacture was based on dye-sensitized solar cells in which the TiO_2_ mesoporous was used as the electron transfer layer. The main reason for using the mesoporous structure was to assume the short diffusion length of the carriers in the perovskite. Researchers later found that the perovskite diffusion length is at least more than 100 nm. Accordingly, TiO_2_ mesoporous is removed from the device structure, and its effect on the performance of the solar cell is investigated in Fig. 2 using the developed modeling. Embedding the mesoporous TiO_2_ in the perovskite-compact TiO_2_ interface can reduce the mobility of charge carriers in this region. This is the reason for obtaining the low charge carriers mobility during the fitting of the simulation results with those of the experimental data (see Fig. 1b and Table S1). Removal of TiO_2_ mesoporous has increased the power conversion efficiency (PCE) from 14.83 to 16.50% due to J_sc_ and FF improvement (Fig. 2a). Investigation of the exciton generation rate profile along the perovskite layer shows that the improved device performance is due to the increased exciton generation rate and charge carrier mobility near compact TiO_2_ (Fig. 2b).

**Figure 3:**
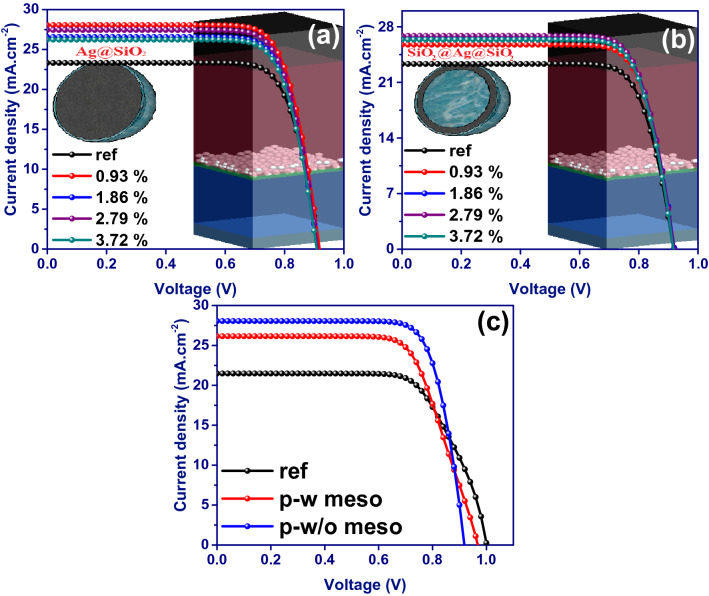
J-V characteristics of the incorporated planar devices with the different mass ratios of (**a**) Ag@SiO_2_ and (**b**) SiO_2_@Ag@SiO_2_ nanospheres. (**c**) J–V characteristics of the device incorporated with a 0.93% mass ratio of Ag@SiO_2_ NPs in the presence (p-w meso) and absence (p-w/o meso) of mesoporous TiO_2_.

To benefit from the plasmonic properties of metallic nanoparticles in improving the performance of carbon-based planer perovskite solar cells, Ag@SiO_2_ core–shell nanoparticles, and SiO_2_@Ag@SiO_2_ triple core–shell nanoparticles have been embedded at the interface of CH_3_NH_3_PbI_3_-compact TiO_2_ (Fig. 3). These plasmonic nanostructures have been designed and optimized in our previous studies and introduced as effective plasmonic nanoparticles^21^. Here, the deformed nanospheres capable of overlapping with each other are randomly distributed in a volumetric space with varying mass ratios (the volume of distributed plasmonic nanoparticles/perovskite layer volume × 100) using a realistic simulation. The overall dimensions of the nanoparticles, the thicknesses of Ag inner shell and SiO_2_ outer shell are 32 nm, 2.5 nm, and 1 nm, respectively.

The device efficiency has increased to 19.72% and 18.20% after adding a 0.93% mass ratio of Ag@SiO_2_ and SiO_2_@Ag@SiO_2_ NPs, respectively, due to the J_sc_ increment shown in Fig. 3. Further increment of the mass ratio of embedded Ag@SiO_2_ nanoparticles decreases the efficiency stemming from the J_sc_ drop (Table 1). This could be due to the enhancement of the reflectivity behavior of nanoparticles, which prevents light from penetrating deep into the perovskite layer. This, in turn, can weaken the performance of the device by reducing the optical absorption. However, the increase in the mass ratio of SiO_2_@Ag@SiO_2_ nanoparticles has increased the device efficiency, and the maximum power conversion efficiency of 18.92% is obtained for the devices incorporated with the mass ratios of 2.79% (Fig. 3b and Table 1). Removal of mesoporous TiO_2_ and adding 0.93% mass ratio of Ag@SiO_2_ NPs between perovskite and compact-TiO_2_ have increased the device efficiency by 33%. The presence of mesoporous TiO_2_ and the addition of NPs within the mesoporous structure could only increase the device efficiency by 17% (Fig. 3c and Table 2). This suggests that the presence of TiO_2_ mesoporous as the surrounding environment of doped plasmonic nanoparticles can weaken their plasmonic enhancement.

**Table 1:** Electrical characteristics of the simulated devices incorporated with SiO_2_@Ag@SiO_2_ and Ag@SiO_2_ NPs with different mass ratios.

NPs	Samples	J_sc_ (mA/cm^2^)	V_oc_ (V)	FF (%)	PCE (%)
Ag@SiO_2_	ref	23.31	0.917	77.10	16.50
	0.93	28.05	0.918	76.57	19.72
	1.86	26.56	0.911	76.65	18.55
	2.79	27.44	0.912	76.55	19.16
	3.72	26.23	0.908	76.66	18.26
SiO_2_@Ag@SiO_2_	ref	23.31	0.917	77.10	16.50
	0.93	25.74	0.920	76.86	18.20
	1.86	26.50	0.920	76.74	18.71
	2.79	26.85	0.919	76.68	18.92
	3.72	26.38	0.916	76.72	18.54

**Table 2:** Electrical characteristics of the simulated device incorporated with a 0.93% mass ratio of Ag@SiO_2_ NPs in the presence (p-w meso) and absence (p-w/o meso) of mesoporous TiO_2_.

NPs	Samples	J_sc_ (mA/cm^2^)	V_oc_ (V)	FF (%)	PCE (%)
w/o doping	ref w meso	21.48	1.00	68.90	14.83
Ag@SiO_2_ (0.93)	p-w meso	26.15	0.968	68.56	17.35
	p-w/o meso	28.05	0.918	76.57	19.72

**Figure 4:**
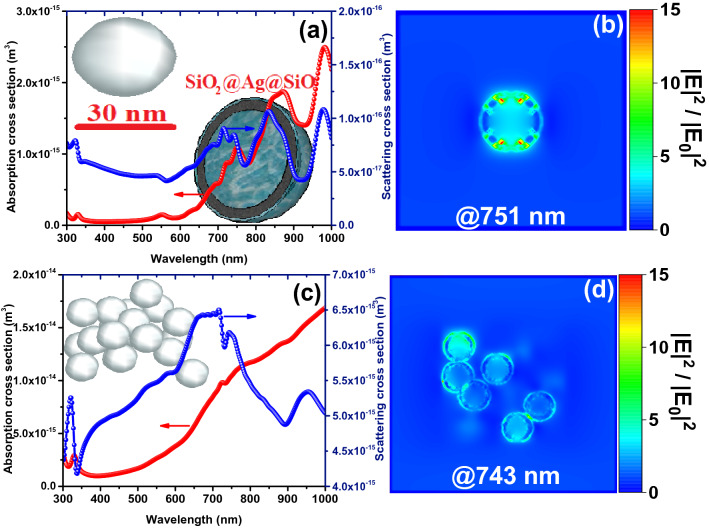
Absorption and scattering cross-sections (**a**, **c**) and near-field intensity enhancement (**b**, **d**) of (**a**, **b**) single and (**c**, **d**) a mass of 20 triple core–shell SiO_2_@Ag@SiO_2_ nanospheres (1 nm SiO_2_ utter shell, 2.5 nm Ag inner shell and total size of 32 nm).

**Figure 5:**
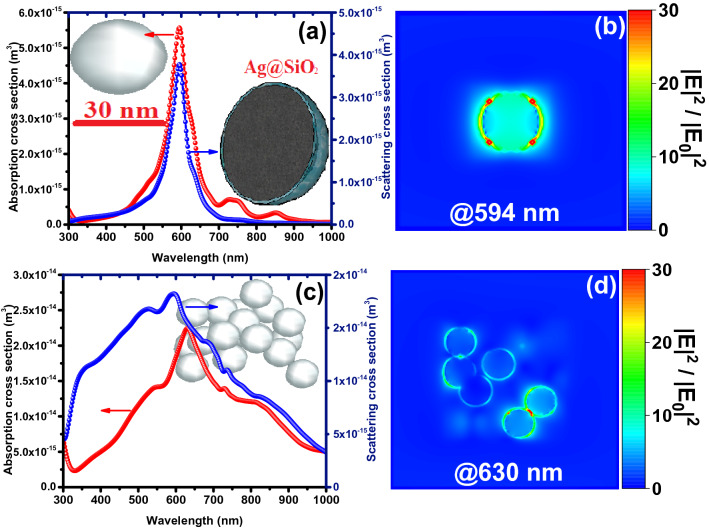
Absorption and scattering cross-sections (**a**, **c**) and near-field intensity enhancement (**b**, **d**) of (**a**, **b**) single and (**c**, **d**) a mass of 20 core–shell Ag@SiO_2_ nanospheres (1 nm SiO_2_ utter shell and total size of 32 nm).

In Figs. 4 and 5, the LSPR properties of SiO_2_@Ag@SiO_2_ and Ag@SiO_2_ nanoparticles embedded in the perovskite environment are investigated, respectively, as single-particle and mass during plasmon resonance. Compared to the single nanoparticle, mass nanoparticles show a larger absorption cross-section due to the production of new plasmonic peaks and their increase in spectral width due to plasmonic interactions of neighboring nanoparticles (Fig. 4a and c). This is a reason for further improvement in the efficiency of the device incorporated with SiO_2_@Ag@SiO_2_ nanoparticles by increasing the mass ratio of embedded nanoparticles (Fig. 3b). Plasmon resonance of SiO_2_@Ag@SiO_2_ nanoparticles occurs due to the plasmon hybridization mechanism at higher wavelengths than Ag@SiO_2_ nanoparticles (Figs. 4 and 5), where the photon flux produced by the nanoparticle is unable to transfer electrons from the valance band to the conduction band of perovskite. This results in less plasmonic improvement of the device in the presence of SiO_2_@Ag@SiO_2_ nanoparticles compared to Ag@SiO_2_ nanoparticles. In addition, Ag@SiO_2_ nanoparticles generate stronger plasmonic fields in the perovskite environment compared to SiO_2_@Ag@SiO_2_ nanoparticles. Figure S2 shows the absorption spectrum of perovskite in the presence of a 0.93% mass ratio of Ag@SiO_2_ and SiO_2_@Ag@SiO_2_ nanoparticles compared to the reference device (without nanoparticles). As expected, perovskite absorption in the presence of Ag@SiO_2_ nanoparticles shows further improvement.

The presence of a carbon electrode (work function of ~ 5 eV) as the non-ideal anode and the absence of an electron blocking layer at the perovskite-carbon interface limits the device efficiency by enhancing the probability of electron recombination at the interface. Adding an appropriate interlayer at the perovskite-carbon interface and/or adequate increment of the working function of carbon by using suitable additives/binders in carbon ink to increase the transfer rate of hole carriers while blocking electron carriers are other approaches to improve the performance of carbon-based perovskite solar cells. In metal electrode-based perovskite solar cells, spiro-OMeTAD has been widely used as a representative of hole transport material^41,42^. However, there are significant limitations to the use of this material (and generally organic and conductive polymer-based molecules) in terms of cost and stability^43,44^. In addition, previous studies have shown that spiro-OMeTAD-equipped carbon electrode-based perovskite solar cells offer lower efficiencies than a metal electrode-based device^45^. This is due to the incompatibility of some organic solvents in carbon paste with spiro-OMeTAD, which leads to its degradation^46^. Therefore, the effect of the inorganic CuSCN layer with excellent semiconducting properties such as high hole mobility, wide bandgap, optical transparency, thermal and chemical stability, and solution processability^44,47^ on the performance of the device has been investigated. It should be noted that CuSCN can be deposited at low temperatures using various techniques including spin and spray coatings, doctor Blade, and electro-deposition^44,48–50^.

**Figure 6:**
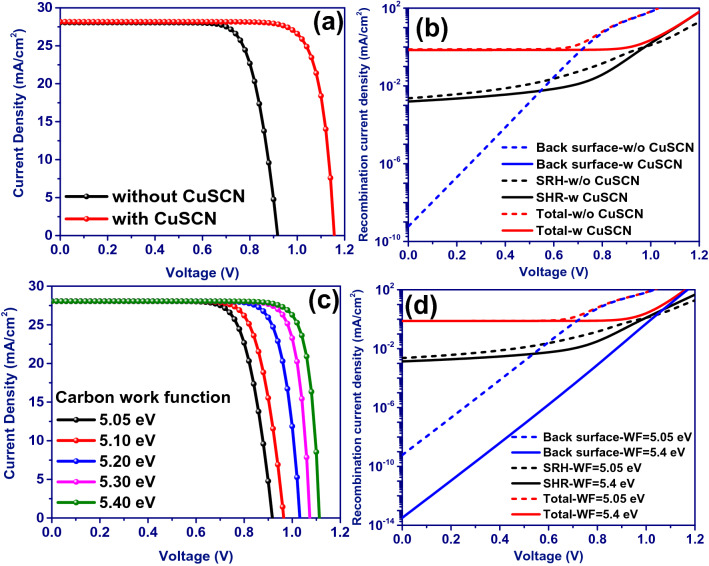
(**a**) J–V characteristics and (**b**) recombination current density of the optimized plasmonic device with and without the CuSCN hole transport layer. (**c**) J–V characteristics and (**d**) recombination current density of the optimized plasmonic device as a function of carbon electrode work function (WF).

Figure 6a shows the J–V characteristic calculated under AM 1.5G irradiation for the device comprised of FTO/TiO_2_/Ag@SiO_2_ NPs: CH_3_NH_3_PbI_3_/CuSCN/Carbon. The optimized plasmonic cell in the absence of the hole transport layer of CuSCN delivers J_sc_ of 28.05 mA/cm^2^, V_oc_ of 0.918 V, FF of 76.57%, and PCE of 19.72%. On the other hand, the used solution-processed inorganic p-type CuSCN layer with a thickness of 30 nm at the perovskite-carbon interface has increased the device PCE by ~ 35% to 26.58% thanks to the increased V_oc_ and FF to 1.156 V and 81.64%, respectively (Table 3). The remarkable improvement of the V_oc_ is attributed to better interfacial energy alignment between the perovskite and CuSCN (see Figure S3). Figure 6b shows the recombination current density for the device equipped with a CuSCN layer compared to the reference device. Adding the CuSCN layer not only has reduced the SRH recombination by producing a back surface field and blocking electrons toward the TiO_2_ ETL but also has reduced the current density of the back surface recombination down to ~ 10^−22^ mA/cm^2^, even at high voltages, by appropriately aligning the interfacial energy.

**Table 3:** Electrical characteristics of the optimized plasmonic simulated device with and w/o the CuSCN hole transport layer and as a function of carbon electrode work function.

Samples		J_sc_ (mA/cm^2^)	V_oc_ (V)	FF (%)	PCE (%)
Adding HTL	Plasmonically optimized	28.05	0.918	76.57	19.72
	Adding CuSCN	28.16	1.156	81.64	26.58
Increasing Carbon work function	Plasmonically optimized (5.05)	28.05	0.918	76.57	19.72
	5.10 eV	28.05	0.964	77.61	20.99
	5.20 eV	28.05	1.032	81.34	23.55
	5.30 eV	28.05	1.074	84.27	25.39
	5.40 eV	28.05	1.113	84.44	26.36

Furthermore, Figs. 6c and d show the performance of the optimized plasmonic device as a function of the back contact work function. As the working function of the carbon electrode increases, which is possible by using suitable additives/binders in the carbon ink, V_oc_ and FF have increased thanks to the excellent alignment of the interfacial energy and the reduction of the recombination current density. As a result, a PCE above 26% is obtained for the work function of 5.4 eV (Table 3).

## Conclusions

In summary, we have investigated the effects of the removal of mesoporous TiO_2_, plasmonic nanoparticle embedding, and utilization of solution-processed inorganic p-type CuSCN as an HTL on the performance of carbon-based PSCs in an electro-optical study. We have shown that removing mesoporous TiO_2_ can increase the device PCE by increasing the exciton generation rate and charge carriers' mobility in the vicinity of the CH_3_NH_3_PbI_3_-compact TiO_2_ interface. Furthermore, we have shown that the embedding of Ag@SiO_2_ and SiO_2_@Ag@SiO_2_ plasmonic nanoparticles can increase the device PCE from 16.50% to 19.72% and 18.92% (in the optimum mass ratio), respectively, thanks to benefiting from the light scattering, trapping, and strong plasmonic fields produced by the nanoparticles. On the other hand, non-ideal carbon electrode has limited the device efficiency by increasing the interface recombination. Adding a solution-processed inorganic p-type CuSCN layer at the perovskite-carbon interface increased the device PCE to 26.58% by significantly improving the V_oc_ and FF thanks to the proper alignment of the interfacial energy and the reduction of the recombination current density. The same results were obtained by increasing the carbon working function (through incorporating suitable additives/binders to the carbon ink), and the cell PCE was enhanced up to 26% in the optimal case. These results pave the ways to achieve high efficiencies in carbon-based PSCs.

## Materials and methods

### Perovskite device fabrication

Firstly, a cleaned FTO glass substrate was treated in TiCl_4_ (40 mM) at 70 °C for 30 min and washed with deionized water. Next, TiO_2_ mesoporous scaffold has been prepared from the mixed TiO_2_ precursor solution including Titanium(IV) ethoxide (Sigma-Aldrich, 12.7 g), concentrated HCl (Merck, 9.7 g), Pluronic P123 (Sigma-Aldrich, 4.0 g), and butyl alcohol (Sigma-Aldrich, 36.3 g). The solution has been dip-coated on the substrate (withdrawing speed; 30 mm/min) and has been subsequently annealed at 450 °C for 1 h. Then, the prepared PbI_2_ solution (1.4 M) via solving PbI_2_ in DMSO/DMF (1:9) has been spin-coated on TiO_2_ mesoporous coated substrate at 2000 rpm for 20 s (during the process, the temperature is kept at 80 °C). To convert the PbI_2_ film to CH_3_NH_3_PbI_3_, CH_3_NH_3_I solution (1 mg mL^−1^) was prepared in a mixed solvent of isopropanol/cyclohexane (1:9), and then, PbI_2_ films were immersed into the CH_3_NH_3_I solution for 12 h, and the as-prepared methylammonium lead triiodide (MAPbI_3_) films have been heated at 100 °C for 15 min^36,37^. Finally, a commercial carbon paste composed of carbon black and graphite has been used for preparing the carbon back electrode. The carbon paste has been painted on the perovskite film followed by spraying an ethanol solution of 1H, 1H,2H,2H-perfluouorodecyltriethoxysilane (Sigma-Aldrich, 5% v/v) on the paste and heating at 100 °C for 1 h^7^.

### Numerical calculations

Optical-electrically coupled simulation regimes have been used to investigate the plasmonic nanoparticles embedding effects on the performance of carbon-based perovskite solar cells. The rate of exciton generation dependent on the absorption profile of the cell was calculated using FDTD software based on solving Maxwell equations^38^. The complex refractive indices obtained from the ellipsometric analyzes of the fabricated device were utilized to simulate and calculate the exciton generation rate profile optically. Perfectly matched layer (PML) boundary conditions are set at the top and bottom of the unit cell along the Z direction. Periodic boundary conditions are located along x and y directions. A plane wave light source propagated along the Z direction is used; the photon flux density is calculated based on standard solar spectrum AM1.5. Experimental data of the materials' complex refractive indices used for optical simulation are shown in Figure S1. To model the electrical response of the cell, the exciton generation rate profile is directly imported into the Solar Cell Capacitance Simulator (SCAPS-1D) software. Photovoltaic measurements of the fabricated devices were utilized to extract electrical parameters by fitting their corresponding *J–V* characteristics. The software uses the finite difference approach to solve electron and hole drift–diffusion equations to describe the motion of charge carriers inside the photoactive layer. Here Shockley–Read–Hall (SRH) model has been used to describe recombination current density^39^. The electrical parameters used for the simulation, which are extracted by fitting the simulation results with experimental data, are shown in Table S1.

## Supplementary Information

Supplementary Information.
