# Supplementary material for: Impacts of plasmonic nanoparticles incorporation and interface energy alignment for highly efficient carbon-based perovskite solar cells
Source: Sci Rep. 2022 Mar 30;12:5367. doi: 10.1038/s41598-022-09284-9 (PMC8967905; doi:10.1038/s41598-022-09284-9)
Supplement: Supplementary file 1 — Supplementary Information. [file 41598_2022_9284_MOESM1_ESM.docx]

Supporting Information

Impacts of Plasmonic Nanoparticles Incorporation and Interface Energy Alignment for Highly Efficient Carbon-Based Perovskite Solar Cells

MirKazem Omrani^1*^, Reza Keshavarzi^2*^, Mojtaba Abdi-Jalebi^3*^, Peng Gao^4*^

^1^Department of Physics, University of Isfahan, Isfahan 81746-73441, Iran

^2^ Department of Chemistry, Catalysis Division, University of Isfahan, Isfahan 81746-73441, Iran

^3^Institute for Materials Discovery, University College London, Malet Place, London WC1E 7JE, United Kingdom

^4^Xiamen Key Laboratory of  Rare Earth Photoelectric Functional Materials, Fujian Institute of Research on the Structure of Matter, Chinese Academy of Sciences, Xiamen 361021, Fujian, People’s Republic of China

^*^Emails: kazem.omrani@semnan.ac.ir (M. K. O.), r.keshavarzi@chem.ui.ac.ir (R. K.), m.jalebi@ucl.ac.uk (M.A.‐J.), peng.gao@firsm.ac.cn (P. G.)

**Figure S1.** Real (n) and Imaginary (k) parts of the dielectric function of CH_3_NH_3_PbI_3_, TiO_2_, CuSCN and FTO layers ^1,2^.

**Table S1.** Simulation parameters obtained from fitting the model to the experimental data and the literatures ^2–5^.

| Parameter | FTO | TiO_2_ | mesoTiO_2_: CH_3_NH_3_PbI_3_ | CH_3_NH_3_PbI_3_ | CuSCN |
| --- | --- | --- | --- | --- | --- |
| Band gap (eV) | **3.2** | **3.1** | **1.55** | **1.55** | **3.5** |
| Electron affinity (eV) | **4.2** | **4.1** | **3.93** | **3.93** | **1.8** |
| dielectric permittivity (relative) | **9.0** | **10.0** | **24.1** | **24.1** | **10.0** |
| Electron mobility (cm^2^.V^-1^.s^-1^) | **2.0e^+1^** | **2.0e^-2^** | **2.0e^-1^** | **1.0** | **1.00e^+2^** |
| Hole mobility (cm^2^.V^-1^.s^-1^) | **1.0e^+1^** | **2.0e^-2^** | **2.5e^-1^** | **2.0e^+1^** | **2.50e^+2^** |
| CB effective density of states (m^-3^) | **1.0e^+19^** | **1.0e^+19^** | **8.1e^+18^** | **8.1e^+18^** | **2.20e^+19^** |
| VB effective density of states (m^-3^) | **1.0e^+19^** | **1.0e^+19^** | **8.1e^+18^** | **8.1e^+18^** | **1.80e^+18^** |
| Doping concentration of acceptors, NA (m^-3^) | **--** | **--** | **1.0e^+9^** | **1.0e^+9^** | **1.00e^+18^** |
| Doping concentration of donors, ND (m^-3^) | **1.0e^+22^** | **5.0e^+16^** | **1.0e^+9^** | **1.0e^+9^** | **--** |

**Figure S2.** Absorption spectrum of perovskite in the presence of a 0.93% mass ratio of Ag@SiO_2_ and SiO_2_@Ag@SiO_2_ nanoparticles compared to the reference device (without nanoparticles).


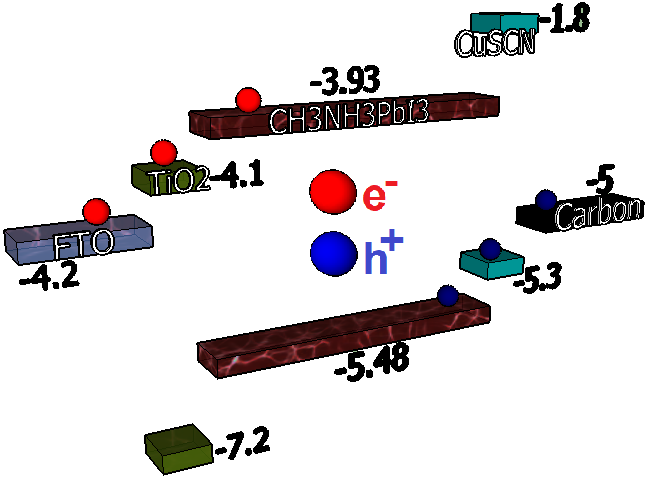


**Figure S3.** Energy level diagram of the device with solution-processed inorganic p-type CuSCN layer.

**References**

1. Kerremans, R. *et al.* On the Electro-Optics of Carbon Stack Perovskite Solar Cells. *Sol. RRL* **4**, 1900221 (2020).

2. Pattanasattayavong, P. *et al.* Electric field-induced hole transport in copper (I) thiocyanate (CuSCN) thin-films processed from solution at room temperature. *Chem. Commun.* **49**, 4154–4156 (2013).

3. Sherkar, T. S., Momblona, C., Gil-Escrig, L., Bolink, H. J. & Koster, L. J. A. Improving perovskite solar cells: insights from a validated device model. *Adv. Energy Mater.* **7**, 1602432 (2017).

4. An, Y. *et al.* Perovskite solar cells: optoelectronic simulation and optimization. *Sol. RRL* **2**, 1800126 (2018).

5. De Los Santos, I. M. *et al.* Optimization of CH3NH3PbI3 perovskite solar cells: A theoretical and experimental study. *Sol. Energy* **199**, 198–205 (2020).
